# Clonal patient-to-patient spread is associated with multidrug-resistant *Acinetobacter baumannii* infections in a Chinese pediatric hospital: an integrated phenotype–genotype–PFGE surveillance study

**DOI:** 10.3389/fmed.2026.1911587

**Published:** 2026-07-22

**Authors:** Wanjun Luo, Xuyang Gong, Sijing Liu, Shiyong Deng, Lei Xi, Changzhen Li, Yuduan Xie, Feng Tang

**Affiliations:** 1 Hospital-Acquired Infection Control Department, Wuhan Children's Hospital (Wuhan Maternal and Child Healthcare Hospital), Tongji Medical College, Huazhong University of Science & Technology, Wuhan, China; 2 Department of Laboratory Medicine, Hubei University of Traditional Chinese Medicine, Wuhan, China; 3 Department of Laboratory Medicine, Wuhan Children's Hospital (Wuhan Maternal and Child Healthcare Hospital), Tongji Medical College, Huazhong University of Science & Technology, Wuhan, China; 4 Children’s Medical Ward I, Wuhan Children’s Hospital (Wuhan Maternal and Child Healthcare Hospital), Tongj Medical College, Huazhong University of Science & Technology, Wuhan, China; 5 Department of Clinical Laboratory, Wangjing Hospital of China Academy of Chinese Medical Sciences, Beijing, China

**Keywords:** *Acinetobacter baumannii*, antimicrobial resistance, biofilm, clonal transmission, OXA-23, pulsed-field gel electrophoresis

## Abstract

**Background:**

Carbapenem-resistant *Acinetobacter baumannii* is a critical-priority pathogen in pediatric hospitals, but the relative roles of clonal transmission and environmental reservoirs remain unclear.

**Methods:**

We analyzed 42 non-duplicate *A. baumannii* isolates collected between 2020 and 2023 from clinical specimens (*n* = 28), medical waste (*n* = 8), and hospital-setting samples (*n* = 6) at Wuhan Children’s Hospital. Antimicrobial susceptibility testing, PCR-based resistance gene detection, pulsed-field gel electrophoresis, and crystal-violet biofilm assays were performed.

**Results:**

Clinical isolates showed high multidrug resistance (75.0%, 21/28), with 75.0% resistant to piperacillin–tazobactam, ceftazidime, imipenem, meropenem, and ciprofloxacin. All isolates remained susceptible to tigecycline. *OXA-23* was detected in all carbapenem-resistant clinical isolates, and *aac(6′)-Ib-cr* was present in all fluoroquinolone-resistant isolates. One dominant clinical clone, T11, accounted for 57.1% of clinical isolates and included one *OXA-23*/*NDM* co-producing isolate. PFGE revealed no clonal overlap between clinical and non-clinical isolates; all medical-waste and hospital-setting isolates were fully susceptible. Biofilm formation was detected in 97.6% of isolates; among clinical isolates, biofilm intensity was inversely associated with multidrug resistance, with susceptible isolates forming stronger biofilm (Fisher’s exact *p* < 0.001).

**Conclusion:**

Pediatric multidrug-resistant *A. baumannii* infections in this hospital were most consistent with patient-to-patient dissemination of a dominant resistant clone rather than with contamination from the hospital-setting or medical-waste reservoirs sampled here, although the small environmental sample cannot definitively exclude such reservoirs. Phenotype–genotype–PFGE surveillance provides actionable guidance for infection control in resource-limited pediatric settings.

## 1 Introduction

*Acinetobacter baumannii* represents one of the most formidable challenges in contemporary infectious-disease medicine. In its 2024 Bacterial Priority Pathogens List, the World Health Organization (WHO) placed carbapenem-resistant *A. baumannii* (CRAB)—not *A. baumannii* as a whole—in the critical-priority tier, the highest of its three tiers, which does not assign individual numerical ranks (WHO Bacterial Priority Pathogens List, 2024). This aerobic, non-fermenting, Gram-negative coccobacillus is a leading cause of healthcare-associated infections worldwide, especially in intensive care units (ICUs), causing ventilator-associated pneumonia, bloodstream infections, and wound sepsis (1). Unlike environmental *Acinetobacter* species from soil and water, it is well adapted to hospital environments, surviving on dry surfaces and equipment in a manner that facilitates nosocomial transmission (2). The 2019 Global Burden of Antimicrobial Resistance (GRAM) study documented 1.27 million deaths directly attributable to bacterial AMR (3), and modelling projects up to 39 million AMR-attributable deaths by 2050 (4). Among the leading resistant pathogens, *A. baumannii* is among the deadliest, with CRAB-attributable mortality of 50–70% versus approximately 25% for carbapenem-susceptible strains (5).

Pediatric patients, particularly those in the PICU and neonatal ICU (NICU), are a uniquely vulnerable group: their immune systems and mucosal barriers are less developed than in adults (6), they are frequently immunocompromised by underlying disease or chemotherapy (7), and they undergo prolonged invasive procedures such as intubation and central venous catheterization. Consequently, once *A. baumannii* invades, disease often progresses more rapidly, and treatment is further constrained by pediatric-specific pharmacokinetics and safety profiles (8, 9).

The molecular arsenal underlying *A. baumannii* resistance includes carbapenem-hydrolyzing class D *β*-lactamases (particularly OXA-23, OXA-24/40, OXA-51, and OXA-58); metallo-β-lactamases (NDM, VIM, IMP); aminoglycoside-modifying enzymes; multidrug efflux pumps (AdeABC, AdeIJK); altered outer-membrane permeability; and target-site modifications (10). Among acquired carbapenemases, OXA-23-like enzymes have achieved global predominance, accounting for 75–88% of CRAB isolates worldwide (5). The OXA-23 gene is frequently associated with insertion sequence IS*Aba*1, which provides strong promoter activity, and mobilization via transposons Tn2006–Tn2008 facilitates horizontal transfer (11).

Biofilm formation represents a critical virulence mechanism enabling *A. baumannii* persistence in healthcare settings. Biofilms confer up to 1,000-fold increased antibiotic tolerance compared with planktonic cells, with minimum biofilm eradication concentrations (MBEC) reaching 44-fold higher for colistin, 407-fold for ciprofloxacin, and 364-fold for imipenem relative to planktonic minimum bactericidal concentrations (12). Global meta-analyses report a biofilm prevalence of 65.6% across diverse clinical settings, with immunocompromised and ICU populations approaching 100% (13, 14).

Molecular epidemiological investigations using PFGE, multilocus sequence typing (MLST), and whole-genome sequencing (WGS) have established that nosocomial *A. baumannii* outbreaks are predominantly driven by dissemination of successful clonal lineages—particularly International Clones IC1 (ST1/ST231), IC2 (ST2/ST208), and IC3 (ST3)—rather than by polyclonal environmental introduction (15). In Chinese pediatric hospitals, a 2021 outbreak investigation in Shanghai identified the dominant clone ST208 producing OXA-23-like carbapenemase in a children’s hospital ICU (16), and a more recent study from southern China reported ST2 dominance (80.5%) with OXA-23 prevalence of 97.6% in pediatric bloodstream infections (17).

Despite extensive characterization of *A. baumannii* in adult ICU settings, comprehensive investigations integrating resistance phenotypes, molecular genotyping, clonal epidemiology, and environmental/medical-waste surveillance in pediatric tertiary hospitals remain limited. The transmission dynamics between clinical isolates and non-clinical reservoirs require elucidation in order to inform targeted infection-control strategies. The present study addresses this gap through multidimensional analysis of *A. baumannii* isolates from three sources—clinical specimens, medical waste, and hospital-setting samples—at a tertiary pediatric center in Wuhan, China. We hypothesized that clonal patient-to-patient transmission, rather than environmental contamination, represents the primary driver of MDR *A. baumannii* infections in this setting.

## 2 Materials and methods

### 2.1 Bacterial isolates and identification

Between 2020 and 2023, a total of 42 non-duplicate *A. baumannii* isolates were collected from Wuhan Children’s Hospital, comprising clinical specimens (*n* = 28), medical waste (*n* = 8), and hospital-setting samples (*n* = 6). Clinical isolates were obtained in 2021–2023 from respiratory tract specimens (tracheal aspirates, sputum), blood cultures, wound swabs, and urine samples from pediatric patients across multiple departments, including the ICU, respiratory medicine, emergency department, and surgical wards. Environmental sampling was conducted on several occasions between December 2020 and November 2021 as part of an infection-control disinfection-assessment programme, with swabs taken both before and after routine disinfection. Hospital-setting sampling targeted high-touch surfaces and shared water points—including bed rails, ventilator controls, infusion pumps, ward door handles, hot-water-dispenser switches, and sink taps—across multiple wards and floors, including the intensive care and surgical intensive care units. The intensive-care units did not yield *A. baumannii*; the six hospital-setting *A. baumannii* isolates were recovered from general-ward high-touch surfaces and water points (a ward door handle, hot-water-dispenser switches, and sink taps). Medical-waste sampling was performed along the hospital’s medical-waste handling and disinfection pathway—surgical-ward corridors, waste-disposal and storage rooms, and cargo elevators—at sequential time points from before disinfection up to 24 h afterwards, yielding eight *A. baumannii* isolates. Per-swab counts for every individual site were not systematically collated in a single register, although the sampled locations, surface types, timing, and disinfection status are specified above. Specimens were inoculated onto blood agar and MacConkey agar plates and incubated at 37 °C for 18–24 h. Suspected colonies exhibiting characteristic morphology (gray-white, smooth, entire edges on blood agar; non-lactose-fermenting on MacConkey agar) were subjected to Gram staining and biochemical testing. Definitive species identification was performed using matrix-assisted laser desorption/ionization time-of-flight mass spectrometry (MALDI-TOF MS, Bruker Daltonics). Spectra were matched against the Bruker MBT reference library (CE-IVD; the versions in routine use at our laboratory during 2020–2023, spanning approximately v10 to v12), and a score of ≥ 2.00 was required for reliable species-level identification, in accordance with the manufacturer’s criteria. *Escherichia coli* ATCC 25922 served as the quality-control strain for antimicrobial susceptibility testing. For conventional PCR, strains previously confirmed to carry each target gene by Sanger sequencing served as positive controls and a no-template reaction served as the negative control; for the real-time carbapenemase assay, the kit’s GAPDH internal control and the positive and negative controls supplied with the kit were included in each run. For PFGE, *Salmonella enterica* serotype Braenderup H9812 served as the molecular-size standard.

### 2.2 Antimicrobial susceptibility testing

Antimicrobial susceptibility was determined by broth microdilution according to CLSI guidelines (M100-Ed35, 2025). Minimum inhibitory concentrations (MICs) were assessed for 10 antimicrobial agents: piperacillin–tazobactam (TZP), ceftazidime (CAZ), cefoperazone–sulbactam (SCF), cefepime (FEP), imipenem (IPM), meropenem (MEM), levofloxacin (LVX), tigecycline (TGC), trimethoprim–sulfamethoxazole (TMP/SMX), and ciprofloxacin (CIP). Bacterial suspensions were prepared to a 0.5 McFarland standard (~1.5 × 10^8^ CFU/mL) in Mueller–Hinton broth, inoculated into commercial microbroth dilution plates, and incubated at 37 °C for 16–20 h prior to MIC determination. Results were interpreted using CLSI M100 breakpoints. Multidrug resistance (MDR) was defined as acquired non-susceptibility to at least one agent in three or more antimicrobial categories per Magiorakos et al. (2).

### 2.3 Resistance gene detection by PCR

The resistance-gene panel was targeted to the acquired determinants most prevalent in *A. baumannii* in China and most directly interpretable against the tested phenotypes, rather than to provide a comprehensive resistome survey. Genomic DNA was extracted using a commercial bacterial DNA extraction kit (TIANamp Bacteria DNA Kit, Tiangen Biotech) following the manufacturer’s protocols. PCR amplification targeted: *β*-lactamase genes (*bla*_TEM_, *bla*_CTX-M_, *bla*_OXA-1_); trimethoprim resistance genes (*dfrA8*, *dfrA14*); sulfonamide resistance genes (*sul2*, *sul3*); and plasmid-mediated quinolone resistance (PMQR) genes (*qnrA*, *qnrB*, *qnrS*, *aac(6′)-Ib-cr*). Primer sequences are listed in Supplementary Table S1. Conventional PCR was performed on a T100 Thermal Cycler (Bio-Rad Laboratories) using previously described primer sets and cycling conditions. Products were visualized by 1.5% agarose gel electrophoresis with EcoDye DNA staining solution (BIOFACT). Carbapenemase genes (KPC, NDM, OXA-23, OXA-48) were detected using a commercial real-time fluorescent PCR kit (Carbapenem Resistance Gene Detection Kit, Fluorescent PCR Method) according to the manufacturer’s instructions. DNA extracted from isolates was added to the reaction mix in a total volume of 25 μL per tube: tube A targeted NDM, OXA-48, and GAPDH (internal control); tube B targeted KPC, OXA-23, and GAPDH. Amplification was performed on an ABI 7500 Real-Time PCR System (Applied Biosystems) under the following conditions: pre-denaturation at 97 °C for 90 s (1 cycle), followed by 40 cycles of denaturation at 97 °C for 2 s and combined annealing/extension at 60 °C for 30 s. GAPDH served as an internal control to monitor specimen quality, transport conditions, nucleic-acid extraction efficiency, and potential PCR inhibition.

### 2.4 Pulsed-field gel electrophoresis (PFGE)

PFGE was performed following the standardized PulseNet protocol of the Chinese Center for Disease Control and Prevention. Bacterial cells were embedded in 1% SeaKem Gold agarose plugs and lysed *in situ*. Genomic DNA was digested with 40 U Apa*I* restriction endonuclease (New England Biolabs) at 37 °C for 4 h. *Salmonella enterica* serotype Braenderup H9812 (digested with Xba*I*) served as the molecular-weight standard. Electrophoresis was conducted using a CHEF Mapper XA System (Bio-Rad) with the following parameters: 6 V/cm, included angle 120°, initial switch time 5 s, final switch time 40 s, total run time 18.5 h at 14 °C in 0.5 × Tris–borate–EDTA (TBE) buffer. Gels were stained with GelRed (Biotium) for 30 min and photographed under UV transillumination. PFGE patterns were analyzed using BioNumerics software v7.6 (Applied Maths). Dendrograms were constructed using the Dice similarity coefficient and the unweighted pair-group method with arithmetic mean (UPGMA). Isolates sharing ≥85% similarity were considered to belong to the same PFGE cluster per Tenover’s interpretive criteria (18).

### 2.5 Biofilm formation assay

Biofilm-forming capacity was quantified using the microtiter plate method with crystal-violet staining, as described by Stepanović et al. (19) Bacterial cultures were adjusted to 0.5 McFarland standard in tryptic soy broth (TSB), further standardized to OD_600_ = 1.0, and 100 μL aliquots were dispensed into 96-well polystyrene plates in four replicate wells within a single experiment. After 24 h incubation at 37 °C, planktonic cells were removed by aspiration, wells were washed three times with sterile distilled water, and biofilms were fixed with 100% methanol for 15 min. After air-drying, biofilms were stained with 0.25% crystal violet for 15 min, excess stain was removed by washing, and the bound dye was solubilized in 33% glacial acetic acid for 5 min with gentle agitation. Optical density was measured at 620 nm (OD_620_) using a microplate reader (Thermo Fisher Scientific). The cut-off OD value (OD_c_) was defined as the mean OD of negative controls plus three standard deviations. Biofilm formation was categorized as: negative (OD ≤ OD_c_), weak positive (OD_c_ < OD ≤ 2 × OD_c_), moderate positive (2 × OD_c_ < OD ≤ 4 × OD_c_), or strong positive (OD > 4 × OD_c_). Inter-replicate reproducibility was summarized as the median coefficient of variation of the OD_620_ readings across isolates.

### 2.6 Statistical analysis

Categorical variables were compared using Fisher’s exact test (for cell counts < 5) or the chi-square test as appropriate. For the biofilm analysis, the replicate optical-density readings of each isolate were averaged to a single value, so that the isolate was the unit of analysis. Because only two intensity categories (weak and moderate) were observed among clinical isolates and one cell of the resulting 2 × 2 table was zero, the association between biofilm-production intensity and the MDR phenotype was evaluated using Fisher’s exact test, and was additionally examined on the continuous OD620 values using the Mann–Whitney *U* test, with the Hodges–Lehmann median difference and its 95% confidence interval reported as the effect size. A two-tailed *p* value < 0.05 was considered statistically significant. Statistical analyses were performed using SPSS version 26.0 (IBM Corporation).

## 3 Results

### 3.1 Antimicrobial susceptibility patterns differ markedly between clinical and non-clinical isolates

**Figure 1:**
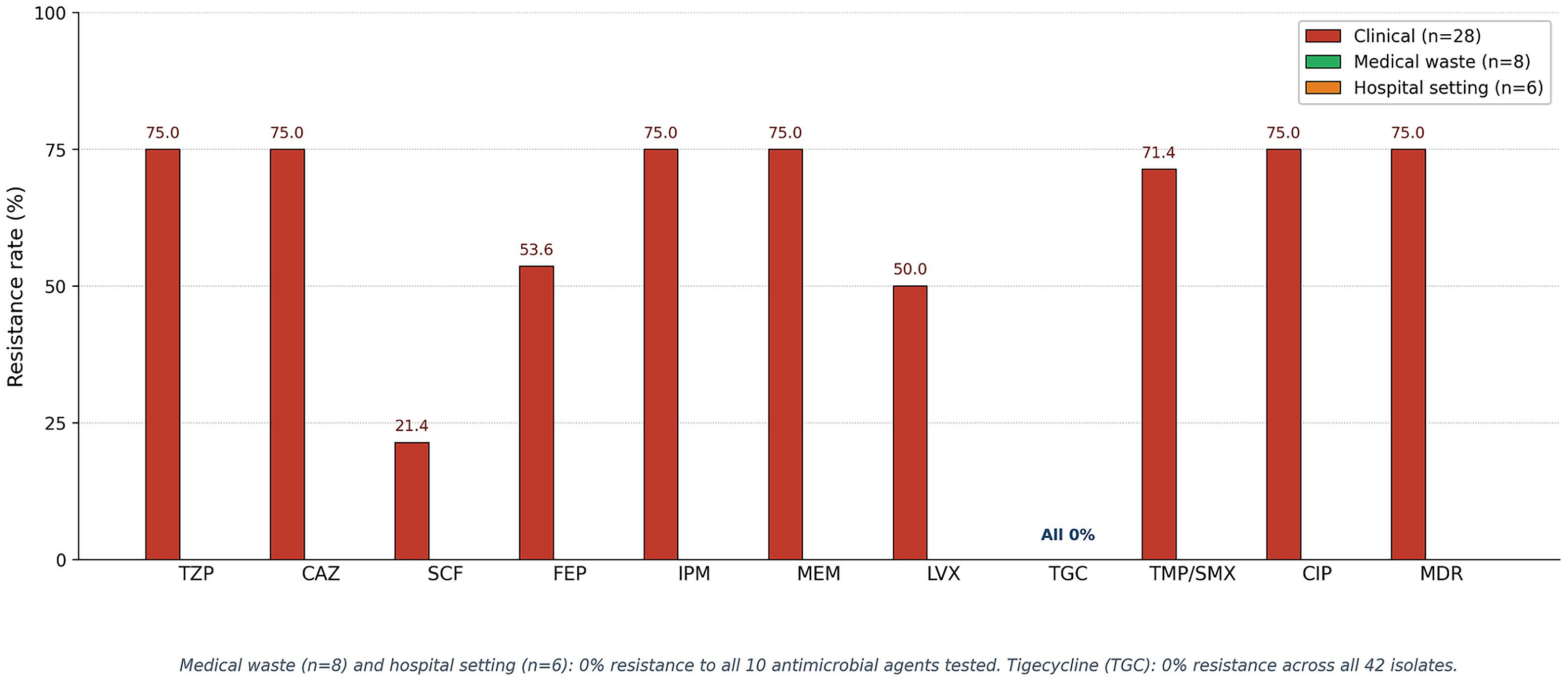
Antimicrobial resistance rates of *Acinetobacter baumannii* isolates, stratified by source. Bar chart displaying the percentage of resistant isolates against each of 10 tested antimicrobial agents and the MDR rate, stratified by isolation source: clinical specimens (*n* = 28), medical waste (*n* = 8), and hospital-setting samples (*n* = 6). Antimicrobial abbreviations are defined in the Abbreviations section. All medical-waste and hospital-setting isolates demonstrated complete susceptibility (0%) to all 10 agents; tigecycline susceptibility was universally preserved across all 42 isolates. Resistance breakpoints were determined per CLSI M100-Ed35 (2025).

**Figure 2:**
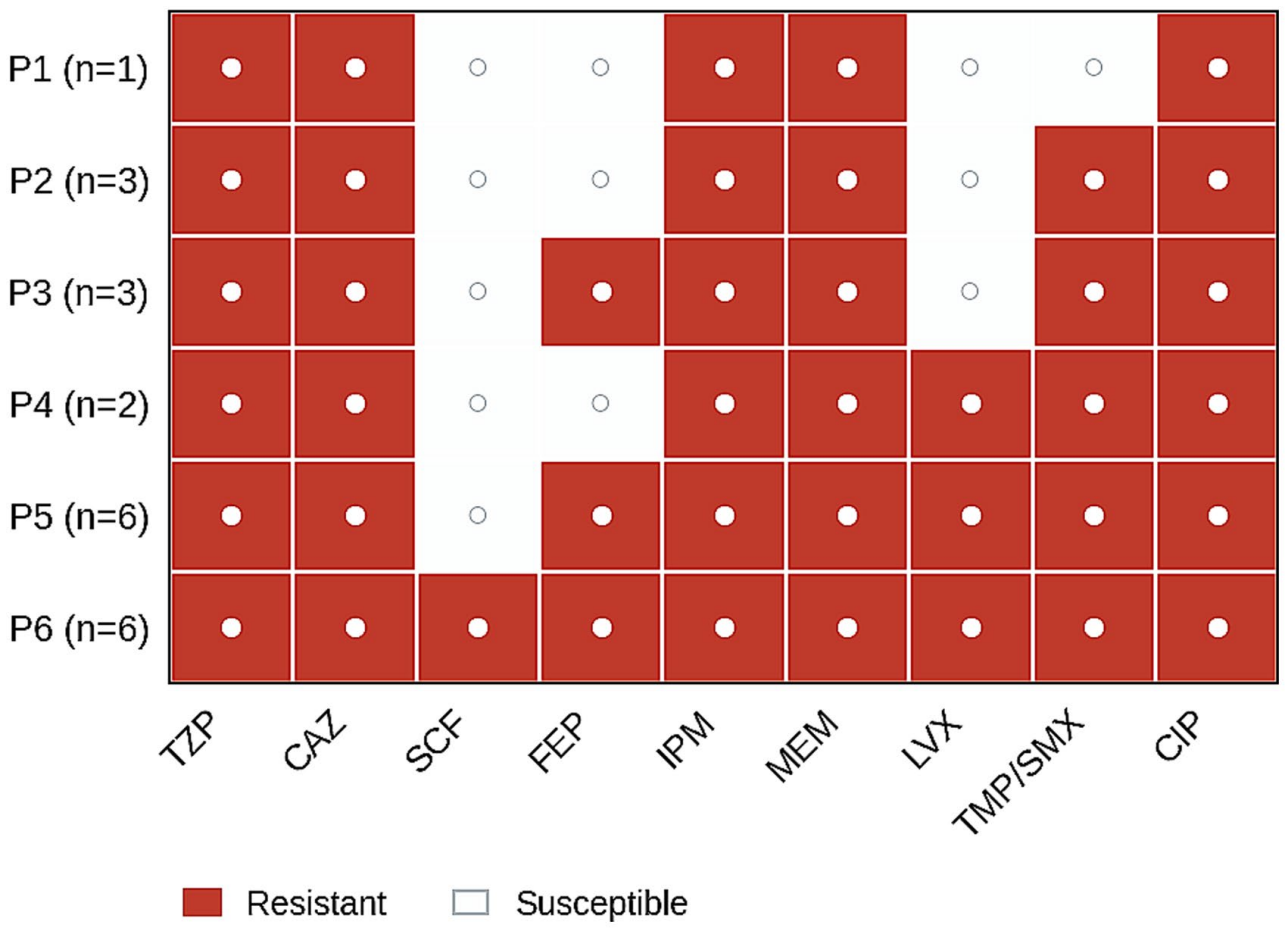
Multidrug-resistance (MDR) profiles of clinical *Acinetobacter baumannii* isolates. Tile-grid representation of the six distinct MDR profiles observed among the 21 MDR clinical isolates (75.0%, 21/28). Each row (P1–P6) corresponds to one resistance profile, ordered by increasing resistance breadth (5- to 9-class). The corresponding PFGE-type designations for the isolates of each profile are listed in Table 1.

Antimicrobial susceptibility testing revealed a clear dichotomy between clinical and non-clinical isolates (Figure 1). All medical-waste (*n* = 8) and hospital-setting (*n* = 6) isolates demonstrated complete susceptibility to all 10 tested antimicrobial agents. In contrast, clinical isolates (*n* = 28) exhibited high-level resistance to multiple antibiotic classes: 75.0% (21/28) were simultaneously resistant to piperacillin–tazobactam, ceftazidime, imipenem, meropenem, and ciprofloxacin. Trimethoprim–sulfamethoxazole resistance was observed in 71.4% (20/28), cefepime resistance in 53.6% (15/28), and levofloxacin resistance in 50.0% (14/28). Cefoperazone–sulbactam resistance was comparatively lower at 21.4% (6/28). Importantly, all 42 isolates—regardless of source—remained susceptible to tigecycline. MDR analysis demonstrated that 75.0% (21/28) of clinical isolates met MDR criteria. The MDR cohort comprised: 1 isolate resistant to 5 drug classes, 3 to 6 classes, 5 to 7 classes, 6 to 8 classes, and 6 to 9 classes, reflecting a spectrum of highly resistant phenotypes (Table 1; Figure 2). The predominant resistance profile was simultaneous resistance to *β*-lactam/β-lactamase inhibitor combinations, third- and fourth-generation cephalosporins, carbapenems, fluoroquinolones, and folate-pathway inhibitors.

**Table 1:** Multidrug-resistance profiles of *Acinetobacter baumannii* clinical isolates (*n* = 21 MDR isolates).

Multidrug resistance profile	Quantity	PFGE type
TZP-CAZ-IPM-MEM-CIP	1	T19-1
TZP-CAZ-IPM-MEM-TMP/SMX-CIP	3	T11-2, T11-3, T12-1
TZP-CAZ-FEP-IPM-MEM-TMP/SMX-CIP	3	T11-6, T11-13, T11-14
TZP-CAZ-IPM-MEM-LVX-TMP/SMX-CIP	2	T11-10, T11-1
TZP-CAZ-FEP-IPM-MEM-LVX-TMP/SMX-CIP	6	T11-4, T11-9, T11-15, T11-16, T13-1, T15-1
TZP-CAZ-SCF-FEP-IPM-MEM-LVX-TMP/SMX-CIP	6	T11-5, T11-7, T11-8, T11-11, T11-12, T22-1

TZP, piperacillin–tazobactam; CAZ, ceftazidime; SCF, cefoperazone–sulbactam; FEP, cefepime; IPM, imipenem; MEM, meropenem; LVX, levofloxacin; TGC, tigecycline; TMP/SMX, trimethoprim–sulfamethoxazole; CIP, ciprofloxacin. PFGE Type denotes the pulsed-field gel electrophoresis pulsotype designation. Strains with the same antibiogram may correspond to different PFGE types and vice versa.

### 3.2 Molecular characterization of Resistance genes

PCR-based resistance gene screening revealed distinct genotypic profiles between resistant clinical isolates and susceptible non-clinical isolates (Table 2). Among β-lactamase genes, *bla*_TEM_ was detected in 46.4% (13/28) of clinical isolates; *bla*_CTX-M_ and *bla*_OXA-1_ were universally absent. The carbapenemase gene OXA-23 was detected in all 21 carbapenem-resistant clinical isolates (21/21, 100%). Real-time fluorescent PCR demonstrated that KPC and OXA-48 were absent from all isolates. NDM co-carriage was identified in one carbapenem-resistant clinical isolate (A18; 1/21, 4.8%), which simultaneously harbored OXA-23, representing a dual-carbapenemase phenotype. The sulfonamide resistance gene *sul2* was detected in only 1/21 (4.8%) resistant isolates; *dfrA8*, *dfrA14*, and *sul3* were absent from all isolates tested. Among non-clinical isolates, resistance genes were largely absent; however, *bla*_TEM_ was detected in one medical-waste isolate (B5), while all six hospital-setting isolates and the remaining seven medical-waste isolates were genotypically susceptible. The PMQR genes *qnrA*, *qnrB* and *qnrS* were absent, whereas *aac(6′)-Ib-cr* was present in all 21 fluoroquinolone-resistant isolates; chromosomal *gyrA* and *parC* mutations were not assessed in this study.

**Table 2:** Detection of *bla_TEM_*, *sul2*, and NDM among 42 *Acinetobacter baumannii* isolates, grouped by detection pattern.

Isolate group	*n*	Origin	*bla_TEM_*	*sul2*	NDM
Hospital-setting isolates (17, 25, 41, 71, 76, A12)	6	Hospital setting	−	−	−
Medical-waste isolates, *bla_TEM_*-negative (B1–B4, B6, B7, B9)	7	Medical waste	−	−	−
B5	1	Medical waste	+	−	−
Susceptible clinical isolates (XL110, XL243, XL244, XL267, XL296, XL346, XL354)	7	Clinical	\	\	\
MDR clinical isolates, *bla_TEM_*-positive (A10, A11, A13, A15, A17, A19, A20, A21, A22, A23, XL252, XL379, XL416)	13	Clinical	+	−	−
MDR clinical isolates, *bla_TEM_*-negative (A14, A16, XL153, XL177, XL386, XL421)	6	Clinical	−	−	−
XL245	1	Clinical	−	+	−
A18 (T11-5)	1	Clinical	−	−	+

+, gene detected; −, gene not detected; \, not tested (isolate phenotypically susceptible; the resistance-gene panel was applied selectively to MDR isolates). All isolate groups shown were fully susceptible (S) except the 21 MDR clinical isolates (rows 5–8), whose individual antibiogram profiles and PFGE types are given in Table 1. bla_CTX-M_, bla_OXA-1_, dfrA8, dfrA14, sul3, qnrA, qnrB, and qnrS were tested but not detected in any of the 42 isolates; KPC and OXA-48 were likewise not detected in any isolate. Aac(6′)-Ib-cr and OXA-23 were detected in all 21 MDR clinical isolates (21/21) and in none of the remaining 21 isolates. Carbapenemase genes (KPC, NDM, OXA-23, OXA-48) were assessed in all 42 isolates by real-time fluorescent PCR; conventional PCR for the remaining genes was applied only to MDR clinical isolates. Isolate A18 (T11-5, highlighted) is the sole NDM co-carrier, within the dominant T11 cluster.

### 3.3 PFGE analysis: dominant clonal transmission without clinical–environmental linkage

**Figure 3:**
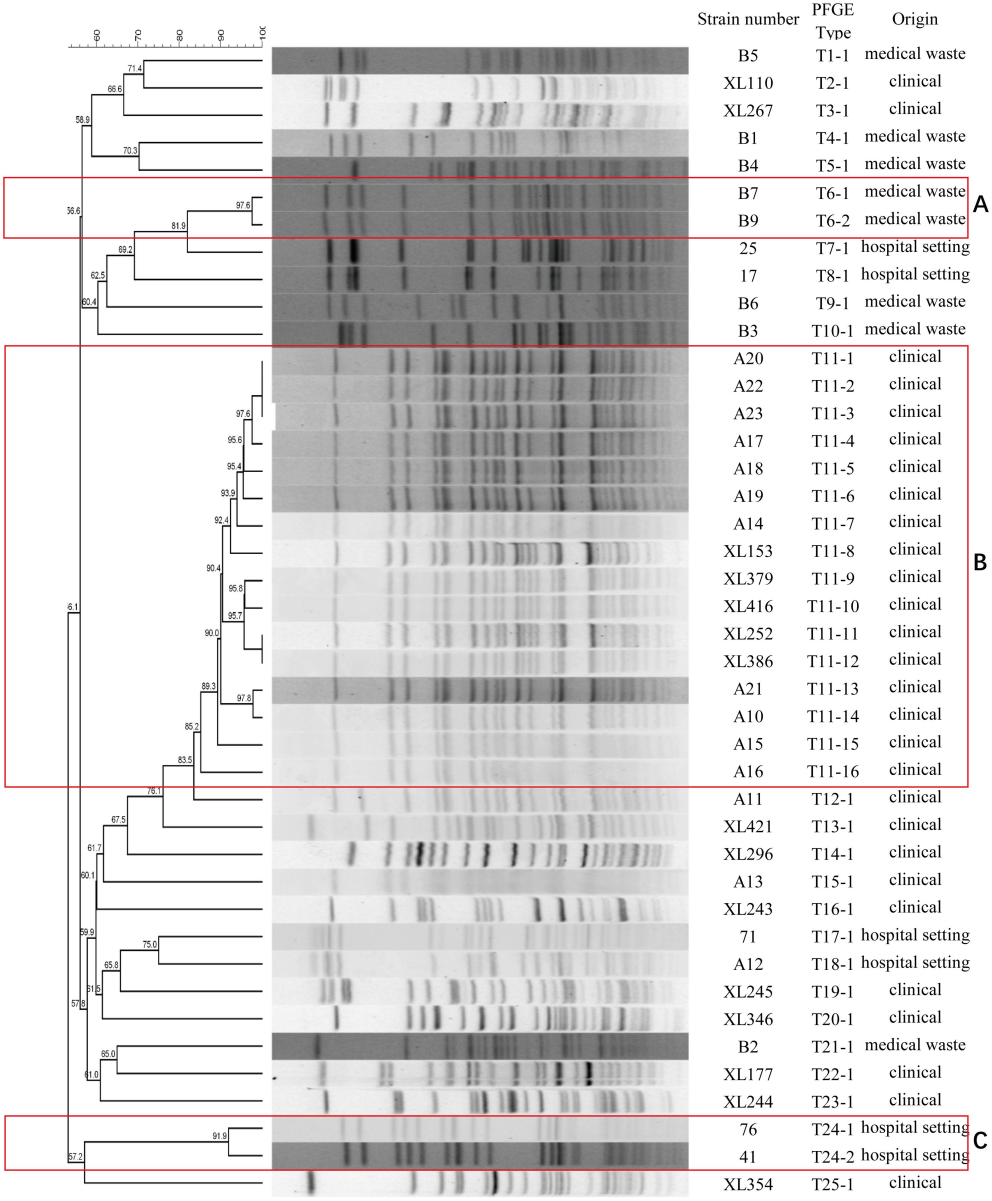
Pulsed-field gel electrophoresis (PFGE) dendrogram of 42 *Acinetobacter baumannii* isolates (ApaI; Dice coefficient; UPGMA; BioNumerics v7.6). Each row represents one isolate, with strain number, PFGE-type designation, and isolation source indicated on the right. Isolates are classified by origin: clinical specimens (*n* = 28), medical waste (*n* = 8), and hospital-setting samples (*n* = 6). Red boxes denote the three multi-isolate clusters: Group A (T6, *n* = 2; medical-waste isolates B7 and B9; 97.6% internal similarity); Group B (T11, *n* = 16; all clinical isolates; ≥90% internal similarity); and Group C (T24, *n* = 2; hospital-setting isolates 76 and 41; 91.9% internal similarity). The remaining 22 isolates each constituted a unique singleton PFGE type, for a total of 25 PFGE types (T1–T25). No clonal overlap was observed between clinical and non-clinical isolates at the 85% similarity threshold.

PFGE analysis of the 42 *A. baumannii* isolates resolved 25 distinct PFGE types (designated T1–T25) at the ≥85% Dice-similarity threshold (Figure 3). Types shared by two or more isolates were designated multi-isolate clusters and labelled Groups A (T6, *n* = 2), B (T11, *n* = 16) and C (T24, *n* = 2); the remaining 22 types were singletons represented by one isolate each. The three clusters and 22 singletons together accounted for all 42 isolates. Group B (T11) was the dominant cluster, encompassing 57.1% (16/28) of all clinical isolates with ≥90% internal genetic relatedness, consistent with a recent common ancestor and, potentially, nosocomial dissemination of a single lineage. All 16 T11 isolates demonstrated the MDR phenotype and harbored both OXA-23 and *aac(6′)-Ib-cr*; notably, one T11 member (A18, designated T11-5) additionally carried NDM, representing a dual-carbapenemase genotype within this dominant cluster. Group A (T6) comprised two medical-waste isolates (B7 and B9; 97.6% similarity), while Group C (T24) comprised two hospital-setting isolates (76 and 41; 91.9% similarity). Critically, comparative PFGE analysis revealed no clonal overlap between clinical isolates and hospital-setting or medical-waste isolates at the 85% similarity cut-off. Groups A and C were exclusively composed of non-clinical isolates, while Group B (the dominant MDR clone) was restricted entirely to clinical specimens. No multi-isolate cluster contained isolates from more than one source category. This source-specific clustering indicates that, during the study period, the hospital-setting and medical-waste reservoirs sampled in this study showed no clonal overlap with clinical MDR *A. baumannii* isolates; this is consistent with—though not proof of—patient-to-patient transmission as the main route, and the small environmental sample cannot exclude these reservoirs as occasional sources.

### 3.4 Near-universal biofilm formation capacity

**Figure 4:**
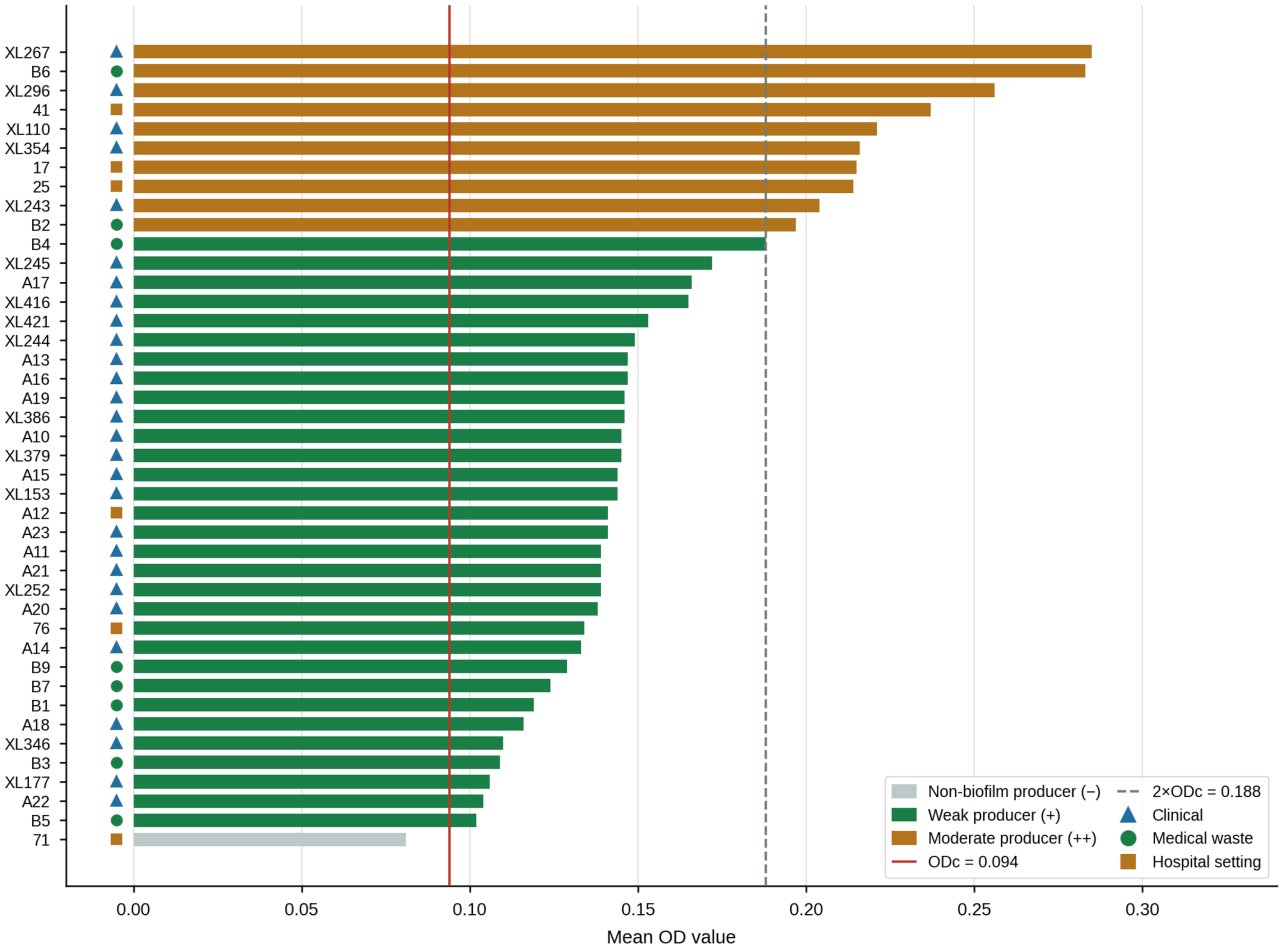
Biofilm-formation capacity of *Acinetobacter baumannii* isolates quantified by crystal-violet microtiter-plate assay. Optical density at 620 nm (OD_620_) values for all 42 isolates are shown, grouped by isolation source. The cut-off (ODc) and categories are as defined in the Methods (ODc = 0.094 and 0.097 for plates 1 and 2). 97.6% of isolates (41/42) exhibited biofilm-forming capacity, predominantly weak-to-moderate producers; the single biofilm-negative isolate (strain 71) originated from a hospital-setting sample. Among clinical isolates, biofilm-production intensity was inversely associated with the MDR phenotype (Fisher’s exact test, *p* < 0.001).

Quantitative biofilm assays demonstrated that 97.6% (41/42) of isolates possessed biofilm-forming capacity (Figure 4). The distribution comprised: 73.8% (31/42) weak producers, 23.8% (10/42) moderate producers, 0% strong producers, and 2.4% (1/42) non-producers. The single biofilm-negative isolate (strain 71) originated from a hospital-setting sample. Across all 42 isolates, the median inter-replicate coefficient of variation of the OD_620_ readings was 10.6% (interquartile range 7.7–13.1%). Replicate wells were averaged to a single optical-density value per isolate, so the unit of analysis was the isolate (*n* = 28 clinical isolates), which fell into only two intensity categories (weak and moderate). Because nearly all isolates were producers (97.6%), a producer-versus-non-producer comparison was uninformative; our analysis was therefore based on biofilm intensity (weak versus moderate), which retained discriminatory power. Contrary to our initial expectation, biofilm-production intensity was inversely associated with the MDR phenotype: all five moderate producers were antimicrobial-susceptible, whereas all 21 MDR isolates were weak producers (Fisher’s exact test, *p* < 0.001). This inverse relationship was confirmed on the continuous OD_620_ values, with susceptible isolates forming significantly more biofilm than MDR isolates (median OD620 0.216 [IQR 0.176–0.238] vs. 0.144 [IQR 0.139–0.147]; Hodges–Lehmann difference 0.072, 95% CI 0.010–0.111; Mann–Whitney *U* test, *p* = 0.008; rank-biserial *r* = 0.69). Because this association rests on a small number of susceptible clinical isolates (*n* = 7) and only two biofilm categories, it should be interpreted with caution.

## 4 Discussion

This molecular epidemiological investigation provides insights into the transmission dynamics, resistance mechanisms, and infection-control implications of *A. baumannii* in a pediatric hospital setting. Our findings are most consistent with MDR *A. baumannii* infections at this institution arising predominantly, during the study period, from patient-to-patient clonal dissemination of a dominant resistant lineage rather than from the environmental reservoirs we sampled; however, the small, cross-sectional environmental sample cannot exclude a contribution from environmental sources. The 75.0% MDR rate among clinical isolates is consistent with the high rates of carbapenem and multidrug resistance reported for *A. baumannii* in recent global surveillance, where regional CRAB prevalence ranges from above 90% in Mediterranean countries to below 1% in Northern Europe, positioning our findings within the intermediate-to-high resistance range (5). Within Chinese pediatric hospitals, a 2021 outbreak in a Shanghai children’s hospital identified ST208 (Oxford scheme) as the dominant CRAB clone producing OXA-23-like carbapenemase (16), while a 2025 study from southern China reported OXA-23 prevalence of 97.6% in pediatric *A. baumannii* bloodstream infections (17). Our 100% (21/21) OXA-23 prevalence in carbapenem-resistant isolates is consistent with this epidemiological landscape.

The universal tigecycline susceptibility observed in our cohort represents a therapeutic opportunity in a setting where carbapenem, cephalosporin, and fluoroquinolone resistance is near-saturating. Tigecycline inhibits bacterial protein synthesis by binding to the 30S ribosomal subunit and overcomes Tet(A)/Tet(B)-mediated efflux and ribosomal protection mechanisms that confer tetracycline resistance (20). Global surveillance indicates tigecycline resistance rates remain below 5.5% in most Asian countries (21). Because this is a susceptibility-based epidemiological study without pharmacokinetic/pharmacodynamic or clinical-outcome data—particularly in children—these findings should be interpreted as showing that tigecycline retained *in vitro* activity against all isolates in this collection, rather than as support for tigecycline as a first-line agent. However, strict antimicrobial stewardship is imperative: tigecycline resistance can emerge through transferable *tet*(X)-family genes and overexpression of AdeABC/AdeIJK efflux pumps, necessitating ongoing susceptibility monitoring (20, 21).

The dichotomy between universally resistant clinical isolates and universally susceptible hospital-setting/waste isolates is noteworthy. The hospital environment is a well-recognized reservoir for *A. baumannii* and has repeatedly been the source of ICU and neonatal outbreaks (22, 23); our failure to detect such a reservoir therefore runs counter to the broader literature and must be weighed against our limited sampling. This contrasts with reports from Iran documenting 84.6% MDR among environmental *A. baumannii* isolates (24) and with a 2025 Shanghai multi-hospital study demonstrating ST2/ST208 (a lineage to which T11 may correspond, although this is unconfirmed pending MLST/WGS) in both clinical and environmental isolates from ICU surfaces and healthcare workers’ hands (25). Our divergent finding may reflect differences in disinfection intensity, restricted high-touch sampling, the small environmental sample (*n* = 6) collected non-systematically on a few occasions in 2020–2021, and limited antibiotic pressure in non-clinical spaces. These factors should be examined prospectively through longitudinal environmental sampling and concurrent antibiotic-use surveillance.

The 100% prevalence of OXA-23 carbapenemase in carbapenem-resistant isolates confirms this enzyme as the predominant carbapenem-resistance mechanism in our setting—consistent with global epidemiology showing OXA-23 accounts for 75–88% of CRAB worldwide (5). OXA-23 belongs to class D *β*-lactamases and demonstrates substrate specificity for penicillins and carbapenems (imipenem > meropenem), with limited activity against extended-spectrum cephalosporins—explaining the 75.0% imipenem/meropenem resistance despite only 53.6% cefepime resistance in our cohort (10).

Of particular clinical significance is the detection of NDM co-carriage in isolate A18 (1/21, 4.8%), which simultaneously harbored OXA-23, representing a dual-carbapenemase phenotype (OXA-23 + NDM). NDM belongs to the Ambler class B metallo-β-lactamase (MBL) family and hydrolyzes virtually all β-lactam antibiotics, including carbapenems, with the notable exception of aztreonam (26). The concurrent presence of OXA-23 and NDM in a single isolate is alarming, as each enzyme independently mediates carbapenem hydrolysis through distinct mechanisms, with no currently available inhibitor active against both simultaneously. Such dual-carbapenemase producers have been increasingly reported globally, including in China, and typically confer higher-level carbapenem resistance and reduced susceptibility to combination therapies (27, 28). Critically, PFGE placed A18 within the dominant T11 cluster (designated T11-5), indicating that this dual-carbapenemase genotype has entered an actively circulating clone, raising the possibility of intra-clonal spread of the NDM-encoding element. Heightened contact isolation of T11-positive patients, systematic carbapenemase-gene monitoring across the T11 population, and WGS characterization of the NDM-carrying plasmid are warranted. Aztreonam–avibactam combinations may offer activity against OXA-23 + NDM co-producers; however, MIC data specific to this isolate would be required before clinical application (29). *aac(6′)-Ib-cr* was detected in all 21 fluoroquinolone-resistant isolates, identifying this plasmid-mediated aminoglycoside acetyltransferase variant as a contributing quinolone-resistance determinant in this cohort. *Aac(6′)-Ib-cr* reduces fluoroquinolone susceptibility approximately 4-fold through acetylation of the piperazinyl ring substituent, a mechanism independent of chromosomal target-site modifications (30). Because this approximately 4-fold effect alone is insufficient to account for high-level resistance, and because chromosomal *gyrA* and *parC* mutations—the principal drivers of fluoroquinolone resistance in *A. baumannii*—were not examined in this study, these mutations are likely the major determinants of the observed phenotype, with *aac(6′)-Ib-cr* acting as an additional contributor. Its co-occurrence with AdeABC/AdeIJK multidrug efflux pumps may produce synergistic reductions in fluoroquinolone susceptibility, a mechanistic interaction warranting efflux-pump expression analysis in future investigations (31). The 46.4% *bla*_TEM_ prevalence approximates Chinese national surveillance data (57.5%), contributing to broad-spectrum penicillin resistance (32).

PFGE analysis is most consistent with nosocomial MDR *A. baumannii* infections in our setting arising from clonal expansion and patient-to-patient transmission rather than from polyclonal environmental introduction; however, PFGE alone cannot establish direct transmission and cannot distinguish it from the circulation of closely related strains, and lacks the resolution to infer high-resolution evolutionary relationships or to assign T11 to an internationally recognized clone (IC1, IC2 or IC3) without MLST or WGS. The dominant T11 cluster, comprising 57.1% of clinical isolates, is consistent with the spread of a successful, hospital-adapted clone. This predominance parallels international surveillance identifying IC2/ST2 in 81.7% of bloodstream-infection isolates in China (33) and ST208 in 93.4% of CRAB isolates in a Chinese children’s hospital outbreak (16). By contrast, environmental sources have been directly implicated in other pediatric *A. baumannii* outbreaks—contaminated incubators (34) and aerosols and air-conditioning systems in a nursery (35)—indicating that clonal patient-to-patient spread and environmental contamination are not mutually exclusive; our negative environmental finding may thus reflect sampling scope rather than the true absence of an environmental contribution.

The absence of clinical–environmental clonal overlap is consistent with reports that hospital-setting strains often differ genetically from patient isolates (24); however, the 2025 Shanghai study showing ST2/ST208 on ICU surfaces and staff hands (25), which indicates such overlap does occur elsewhere, and our contrasting result must be read against the small, cross-sectional environmental sample, which—as in a Latvian NICU outbreak where repeated high-touch surface cultures were negative despite a genuine environmental (ventilation) source (23)—can fail to detect a true reservoir. Longitudinal environmental sampling concurrent with clinical surveillance is needed to define the role of environmental reservoirs at our institution.

The absence of clonal overlap across sources indicates that targeted infection-control interventions should prioritize interruption of patient-to-patient transmission: hand-hygiene compliance, contact-precaution adherence, single-patient equipment designation, and active surveillance cultures for T11-clone carriers in high-risk departments (NICU, PICU, respiratory wards). Multimodal bundles of this type have achieved sustained CRAB elimination without ward closure (36). The 97.6% biofilm-formation prevalence exceeds the global pooled estimate of 65.6% (13) but aligns with investigations focused on ICU and immunocompromised populations (14). The predominance of weak-to-moderate producers suggests that strong-biofilm phenotypes may incur fitness costs, or that 24-h static assays under-represent *in vivo* maturation.

In our cohort, biofilm-production intensity was inversely associated with the MDR phenotype: susceptible clinical isolates formed significantly more biofilm than MDR isolates (Fisher’s exact *p* < 0.001; Mann–Whitney *p* = 0.008 on continuous OD_620_ values). This direction is opposite to reports describing a positive biofilm–MDR correlation in *A. baumannii* (37), but is compatible with a fitness trade-off, in which the metabolic and regulatory cost of maintaining multiple resistance determinants may be offset by reduced investment in biofilm-associated phenotypes. Several caveats temper this interpretation: the finding rests on only seven susceptible isolates and a narrow range of biofilm intensities (only weak and moderate categories were observed among clinical isolates); the crystal-violet static assay does not capture dynamic biofilm maturation; and the *gyrA/parC* and *adeABC/adeIJK* backgrounds that may jointly shape both resistance and biofilm were not characterized. The inverse association therefore requires confirmation in larger cohorts using dynamic biofilm models. Regardless of its relationship to the MDR phenotype, the near-universal biofilm capacity has direct clinical implications: biofilm-embedded organisms demonstrate 44- to 407-fold increases in minimum biofilm eradication concentrations relative to planktonic minimum bactericidal concentrations (12), rendering standard antibiotic dosing insufficient and necessitating biofilm-disrupting adjuvants and early removal of unnecessary invasive devices.

The resistance phenotype–genotype–PFGE surveillance strategy provides a feasible framework for MDR pathogen monitoring in resource-constrained settings. PFGE retains advantages for institutional outbreak response: results within 48–72 h, established discriminatory power, and independence from bioinformatics infrastructure (38). While WGS offers superior resolution and should be the target platform for future studies, PFGE with resistance-gene typing provides actionable information for acute infection-control response where WGS is unavailable.

Taken together, these findings support a targeted control framework: active T11 surveillance with single-room isolation and dedicated equipment in the NICU, PICU and respiratory wards; restricted empirical carbapenem/quinolone use with tigecycline reserved for confirmed MDR infection under quarterly susceptibility monitoring (watching for *tet*(X) and AdeABC efflux); expanded environmental, staff-hand and waste-stream sampling triggered by outbreak signals; and biofilm-directed measures—enhanced device disinfection and minimized indwelling-device duration.

Several limitations warrant explicit acknowledgement. First, the moderate sample size (*n* = 42)—particularly for non-clinical sources (medical waste *n* = 8; hospital setting *n* = 6)—and the single-institution, cross-sectional design limit generalizability and statistical power. The small environmental sample, collected non-systematically on a few occasions in 2020–2021, may under-detect transient contamination events; accordingly, the absence of clonal overlap between the environmental and clinical isolates sampled here should be interpreted within these sampling constraints and does not exclude environmental reservoirs. Second, detailed patient-level clinical data (age, sex, department, underlying disease, antibiotic-exposure history, indwelling device use, length of stay, ward-transfer history, infection site, clinical outcome) were not collected; case–control analyses comparing patients infected with the dominant T11 clone versus those infected with non-T11 strains would substantially strengthen mechanistic conclusions about transmission and risk factors and should be addressed in future work. Third, MLST typing and WGS were not performed; consequently, the T11 cluster cannot be definitively assigned to an international clone (e.g., ST2/ST208), and resistance plasmid structure, IS*Aba*125 mobile-element context for NDM, and complete resistome profiling could not be characterized. Although NDM was identified in isolate A18 within the dominant T11 cluster, the mode of intra-clonal NDM spread—whether through clonal vertical inheritance or horizontal plasmid transfer—requires WGS-based resolution. Fourth, PFGE provides limited resolution for microevolutionary analysis and cannot distinguish between transmission and convergent emergence of similar pulsotypes. Fifth, our resistance-gene panel targeted established determinants and did not comprehensively survey novel or rare mechanisms, including rare carbapenemase variants (IMP, VIM, KPC, OXA-24/40, OXA-58), IS*Aba*1 insertion sequences upstream of *bla*_OXA-23_, transferable *tet*(X) genes, or chromosomal *gyrA*/*parC* mutations contributing to fluoroquinolone resistance. Sixth, healthcare-worker-hand sampling, which is increasingly recognized as an important reservoir, was not performed. Each of these limitations directly motivates the future-work agenda described above. Finally, several subgroups were small—most notably the environmental sources (hospital setting, *n* = 6; medical waste, *n* = 8) and the susceptible clinical isolates (*n* = 7)—so non-significant comparisons should be interpreted with caution given the increased risk of Type II error, and the significant inverse biofilm–MDR association, although large in magnitude, likewise requires confirmation in larger cohorts. In addition, precise per-isolate collection dates were not available at sufficient granularity to reconstruct the temporal dynamics of individual clones; consequently, we could not determine whether the dominant T11 clone persisted throughout the clinical sampling period or reflected a limited outbreak, which would require prospective, date-stamped surveillance with WGS.

## 5 Conclusion

This single-center molecular epidemiological investigation indicates that, at Wuhan Children’s Hospital between 2020 and 2023, MDR *A. baumannii* infections were most consistent with clonal patient-to-patient dissemination of a dominant resistant lineage (T11 cluster) rather than with contamination from the environmental or medical-waste reservoirs sampled in this study. Given the single-center design and the limited environmental sample, and the limited resolution of PFGE (which cannot distinguish direct patient-to-patient transmission from the circulation of closely related strains), these findings are specific to this setting and require confirmation—including by MLST/WGS—before broader generalization. The ubiquitous OXA-23 carbapenemase (21/21 carbapenem-resistant isolates), the detection of NDM co-carriage within the dominant T11 clone (isolate A18, T11-5), and *aac(6′)-Ib-cr* universality in resistant isolates, combined with preserved tigecycline susceptibility across all 42 isolates, provides actionable therapeutic and infection-control guidance while underscoring antimicrobial-stewardship imperatives. The genetic divergence between resistant clinical isolates and susceptible non-clinical isolates is consistent with current infection-control practices, and emphasizes that interventions must focus on interrupting patient-to-patient transmission pathways. Near-universal biofilm-forming capacity underscores the pathogen’s persistence and treatment challenges. The integrated resistance phenotype–genotype–PFGE surveillance framework offers a practical, resource-appropriate approach for outbreak detection and intervention optimization in pediatric healthcare settings where WGS infrastructure may be unavailable. Sustained institutional vigilance, rigorous infection-control adherence, judicious antimicrobial use, and expansion toward MLST/WGS-based surveillance are critical to preventing further escalation of this public-health threat in vulnerable pediatric populations.

## Data Availability

The original contributions presented in the study are included in the article/Supplementary material, further inquiries can be directed to the corresponding authors.

## Glossary

*aac(6′)-Ib-cr*: aminoglycoside acetyltransferase (6′)-Ib-cr
ADC: *Acinetobacter*-derived cephalosporinase
*AdeABC / AdeIJK*: resistance–nodulation–division (RND)-type multidrug efflux pumps
AMR: antimicrobial resistance
ATCC: American Type Culture Collection
*bla*: *β*-lactamase-encoding gene (e.g., *bla*TEM, *bla*CTX-M, *bla*OXA)
CAZ: ceftazidime
CFU: colony-forming unit
CHEF: contour-clamped homogeneous electric field
CI: confidence interval
CIP: ciprofloxacin
CLSI: Clinical and Laboratory Standards Institute
CRAB: carbapenem-resistant *Acinetobacter baumannii*
*dfrA*: dihydrofolate reductase gene
DNA: deoxyribonucleic acid
EDTA: ethylenediaminetetraacetic acid
ESBL: extended-spectrum *β*-lactamase
EUCAST: European Committee on Antimicrobial Susceptibility Testing
FEP: cefepime
GAPDH: glyceraldehyde-3-phosphate dehydrogenase
GRAM: Global Research on Antimicrobial Resistance
*gyrA*: DNA gyrase subunit A gene
IC: international clone
ICU: intensive care unit
IMP: imipenemase-type metallo-*β*-lactamase
IPM: imipenem
*ISAba1*: insertion sequence Aba1
KPC: *Klebsiella pneumoniae* carbapenemase
LVX: levofloxacin
MALDI-TOF MS: matrix-assisted laser desorption/ionization time-of-flight mass spectrometry
MBEC: minimum biofilm eradication concentration
MBL: metallo-β-lactamase
MDR: multidrug-resistant
MEM: meropenem
MIC: minimum inhibitory concentration
MLST: multilocus sequence typing
NDM: New Delhi metallo-β-lactamase
NICU: neonatal intensive care unit
OD_620_: optical density at 620 nm
OD_c_: cut-off optical density
OXA: oxacillinase-type carbapenemase
*parC*: topoisomerase IV subunit C gene
PCR: polymerase chain reaction
PER: Pseudomonas extended-resistance *β*-lactamase
PFGE: pulsed-field gel electrophoresis
PICU: pediatric intensive care unit
PMQR: plasmid-mediated quinolone resistance
*qnr*: quinolone-resistance (pentapeptide-repeat) gene
SCF: cefoperazone–sulbactam
ST: sequence type
*sul*: sulfonamide-resistance gene
TBE: Tris–borate–EDTA
*tet*: tetracycline-resistance gene [*tet*(A), *tet*(B), *tet*(X)]
TGC: tigecycline
TMP/SMX: trimethoprim–sulfamethoxazole
TSB: tryptic soy broth
TZP: piperacillin–tazobactam
UPGMA: unweighted pair-group method with arithmetic mean
VIM: Verona integron-encoded metallo-β-lactamase
WGS: whole-genome sequencing
WHO: World Health Organization
