# Supplementary material for: Clonal patient-to-patient spread is associated with multidrug-resistant Acinetobacter baumannii infections in a Chinese pediatric hospital: an integrated phenotype–genotype–PFGE surveillance study
Source: Front Med (Lausanne). 2026 Jul 22;13:1911587. doi: 10.3389/fmed.2026.1911587 (PMC13439404; doi:10.3389/fmed.2026.1911587)
Supplement: Supplementary file 1 [file Table_1.DOCX]

**Supplementary Table S1** Gene targets and primer sequences used in this study for detection of antimicrobial resistance genes and quinolone resistance-associated mutations.

| **Gene** | **Primer sequence (5′–3′)** | **Amplicon size (bp)** | **Annealing temperature (°C)** |  |
| --- | --- | --- | --- | --- |
| **β-Lactams** | | | | |
| *bla_CTX-M_* | ATGTGCAGYACCAGTAARGT  TGGTRAARTARGTSACCAGA | 593 | 50 |  |
| *bla_TEM_* | ATGAGTATTCAACTTTCCGTGT  TTACCAATGCTTAATCAGTGACG | 876 | 50 |  |
| *bla_OXA_* | ACACAATACATATCAACTTCGC  AGTGTGTTTAGAATGGTGATC | 885 | 50 |  |
| **Sulfonamides** | | | | |
| *sul2* | TCAACATAACCTCGGACAGT  GATGAAGTCAGCTCCACCT | 707 | 50 |  |
| *sul3* | GAGCAAGATTTTTGGAATCG  CATCTGCAGCTAACCTAGGGCTTGGA | 799 | 55 |  |
| **Trimethoprim** | | | | |
| *dfrA8* | GAGCTTCCGGGTGTTCGTGAC  CTTCCATGCCATTCTGCTCGTAGT | 247 | 56 |  |
| *dfrA14* | TTAACCCAGGATGAGAACCT  CGATTGCATAGCTTTGTTAA | 510 | 56 |  |
| **Quinolones** | | | | |
| *aac(6′)-1b-cr* | GCAACGCAAAAACAAAGTTAGG  GTGTTTGAACCATGTACA | 560 | 50 |  |
| *qnrA* | GATAAAGTTTTTCAGCAAGAGG  ATCCAGATCGGCAAAGGTTA | 543 | 53 |  |
| *qnrB* | GATCGTGAAAGCCAGAAAGG  ACGATGCCTGGTAGTTGTCC | 469 | 53 |  |
| *qnrS* | ATGGAAACCTACAATCATAC  AAAACACCTCGACTTAAGT | 492 | 48 |  |
